# Passive mechanical properties of the left ventricular myocardium and extracellular matrix in hearts with chronic volume overload from mitral regurgitation

**DOI:** 10.14814/phy2.15305

**Published:** 2022-07-24

**Authors:** Daniella Corporan, Maher Saadeh, Alessandra Yoldas, Jahnavi Mudigonda, Brooks Alexander Lane, Muralidhar Padala

**Affiliations:** ^1^ Structural Heart Research and Innovation Laboratory Carlyle Fraser Heart Center Emory University Hospital Midtown Atlanta Georgia USA; ^2^ Division of Cardiothoracic Surgery Department of Surgery Emory University School of Medicine Atlanta Georgia USA

**Keywords:** mitral regurgitation, mitral valve prolapse, myocardial mechanics, myocardial remodeling, valvular heart disease

## Abstract

Cardiac volume overload from mitral regurgitation (MR) is a trigger for left ventricular dilatation, remodeling, and ultimate failure. While the functional and structural adaptations to this overload are known, the adaptation of myocardial mechanical properties remains unknown. Using a rodent model of MR, in this study, we discern changes in the passive material properties of the intact and decellularized myocardium. Eighty Sprague‐Dawley rats (350–400 g) were assigned to two groups: (1) MR (*n* = 40) and (2) control (*n* = 40). MR was induced in the beating heart by perforating the mitral leaflet with a 23G needle, and rats were terminated at 2, 10, 20, or 40 weeks (*n* = 10/time‐point). Echocardiography was performed at baseline and termination, and explanted hearts were used for equibiaxial mechanical testing of the intact myocardium and after decellularization. Two weeks after inducing severe MR, the myocardium was more extensible compared to control, however, stiffness and extensibility of the extracellular matrix did not differ from control at this timepoint. By 20 weeks, the myocardium was stiffer with a higher elastic modulus of 1920 ± 246 kPa, and a parallel rise in extracellular matrix stiffness. Despite some matrix stiffening, it only contributed to 31% and 36% of the elastic modulus of the intact tissue in the circumferential and longitudinal directions. At 40 weeks, similar trends of increasing stiffness were observed, but the contribution of extracellular matrix remained relatively low. Chronic MR induces ventricular myocardial stiffening, which seems to be driven by the myocyte compartment of the muscle, and not the extracellular matrix.

## Graphical abstract

Chronic mitral regurgitation causes stiffening of the myocardium, dominated by cardiomyocyte stiffening.

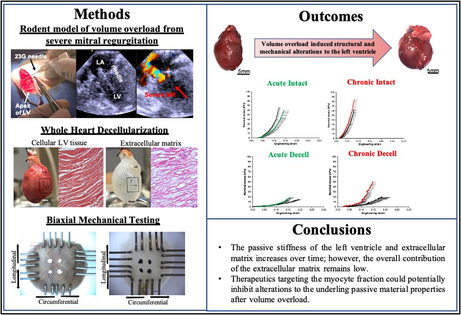

## 1 INTRODUCTION

Mitral regurgitation (MR) is a common heart valve lesion, prevalent in 1.7% of the US population (Benjamin et al., 2019; Nkomo et al., 2006) and the second most common valve complication requiring surgery (Iung et al., 2003). In this valve lesion, leakage of blood from the left ventricle (LV) to the left atrium through the mitral valve in systole, elevates the diastolic inflow and filling volume in the LV in the subsequent cardiac cycle. This increased filling volume stretches the ventricular walls, imposes a volume overload (VO) stress on the heart muscle, and initiates its remodeling (Corporan, Onohara, et al., 2020; Grossman et al., 1975; Kleaveland et al., 1988; Mohamed et al., 2016; Wang et al., 2003). In a rodent model of MR, we recently demonstrated that the dilation and spherical remodeling of the LV is rapid, wall thickness is preserved, functional deterioration measured using ejection fraction with echocardiography is gradual, but load‐independent parameters measured from invasive hemodynamics indicate early contractile dysfunction (Corporan, Onohara, et al., 2020). The mechanistic basis for such remodeling may be due to cardiomyocyte elongation that could increase overall chamber circumference, or extracellular matrix distension to the stress that could increase chamber compliance and slippage of the myocytes resulting in a larger chamber, a combination of both, or just increased elasticity of the chamber that is stretched more in diastole without any permanent changes in the tissue (Corin et al., 1991; De Stefano et al., 2006; McCullagh et al., 1972). Corin et al. (1991) was the first to use invasive hemodynamic measurements in patients with MR to estimate ventricular stiffness, hypothesizing that decreased ventricular stiffness (or increased compliance) as the underlying mechanism of adaptive remodeling in the setting of MR induced VO.

Using non‐valvular VO with arterio‐venous fistula in the rodent model, several studies have demonstrated that elevated ventricular compliance may arise from extracellular matrix degradation that is mediated by inflammatory, protease‐induced remodeling (Chen et al., 2011; Chung & Granzier, 2011; Hutchinson et al., 2010). Loss of collagen and myofiber rearrangement have been demonstrated (Chen et al., 2011; Dell’Italia et al., 1997; Dorn, 2009; Hutchinson et al., 2010; Liu et al., 1991; Ryan et al., 2007), and implicated in the increased compliance. While these studies delineated the structural changes in the extracellular matrix compartment and its potential degeneration in the setting of VO, the extent to which such matrix changes contribute to overall changes in ventricular compliance is not known.

A contrarian hypothesis is that ventricular dilatation occurs in MR without changes in compliance, but via cardiomyocyte elongation from the elevated hemodynamic stress imposed on the walls. The heart consists of layers of myofibers which run‐in opposing directions and angles from the epicardium to the endocardium, and from the base to the apex. These fibers terminate at both their ends in the atrioventricular groove, which is a fibrous structure at the base of the ventricular chambers that acts as an anchor. Within the myofiber, the myofibrils consist of cardiomyocytes whose ends are anchored in series at the gap junction, the sarcomeres within the cells that are anchored in series at their Z‐disks, and Z‐disks that are anchored to titin and other cytoskeletal proteins. According to the law of LaPlace, left ventricular wall stress is directly proportional to changes in chamber pressure, and diameter, and inversely proportional to the wall thickness (Valentinuzzi & Kohen, 2011). When diastolic wall stress is elevated in the setting of VO from MR, longitudinal stretching of all the components, from the myofibers to the cytoskeletal proteins is possible (Ashikaga et al., 2004; Donker et al., 2007; Liu et al., 1991; Yoshida et al., 2010). Badke & Ashikaga reported in dog models of VO that earliest myofiber elongation occurred at the basal aspect of the heart, where they are anchored to the fibrous atrioventricular groove (Badke & Covell, 1979). As remodeling progresses, changes in myofiber angles were observed. In two consecutive studies of chronic VO in rats, an increase in both cardiomyocyte length (from ~120 to ~160 µm), with an increase in cardiomyocyte cross‐sectional area were reported (Gerdes et al., 1988; Liu et al., 1991). It is possible that cardiomyocyte elongation alone can contribute to the left ventricular diameter changes. Elongated cardiomyocytes undergo cytoskeletal remodeling, with changes in the titin protein that spans the Z‐disks, and remodeling of the microtubule network surrounding the sarcomeres. These changes could alter the stiffness of the cardiomyocytes, based on recent studies (Hutchinson et al., 2015; Nishimura et al., 2006; Sequeira et al., 2014; Swiatlowska et al., 2020).

In this study, we sought to quantify the changes in the left ventricular myocardial material properties in the setting of chronic VO from MR. The properties of the intact ventricular myocardium, and the extracellular matrix after removing the myocytes were studied. Biaxial mechanical testing was performed on both tissues to measure changes in the passive material properties, over time, and in comparison, to age‐matched control animals.

## 2 MATERIALS AND METHODS

### 2.1 Ethical statement

The study protocol was reviewed and approved by the Institutional Animal Care and Use Committee at Emory University, and the study was conducted following the NIH guidelines for use of animals in research. Animals were purchased from a single vendor, Envigo, and housed at an Association for Assessment and Accreditation of Laboratory Animal Care (AAALAC) accredited facility.

### 2.2 Animal selection and experimental design

Adult, male, Sprague‐Dawley rats (*n* = 80, 350–400 g, 12–14 weeks) were acquired and housed in cages in a temperature and humidity‐controlled environment (20°C) with 12:12 h light‐dark cycle. Rats had continuous access to standard rodent chow (Teklad Certified Rodent Diet) and drinking water. Rats were assigned to two groups‐ VO from MR (*n* = 40), and control rats without MR that were healthy, age‐matched (*n* = 40). In each group, 10 rats were terminated at 2, 10, 20, and 40 weeks, with left ventricular function measurements using echocardiography and quantification of biaxial myocardial material properties performed at each time point.

### 2.3 Surgical procedure to induce MR

**FIGURE 1:**
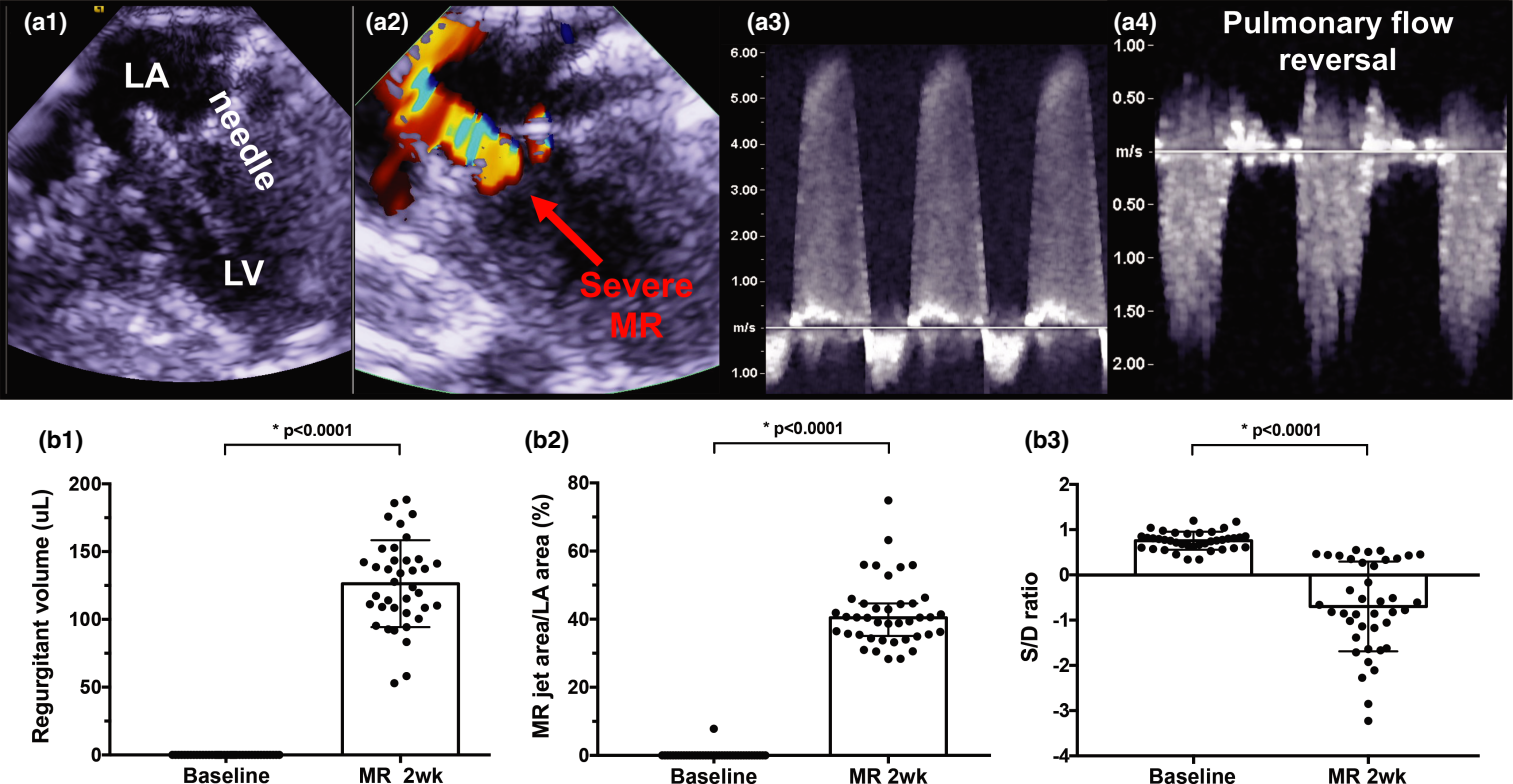
Creation and validation of volume overload induced severe mitral regurgitation. (a1) Two‐chamber transesophageal echocardiographic view visualizing the 23G needle perforating the mitral leaflet; (a2) color Doppler image indicated the MR jet; (a3) MR jet visualized on a pulsed‐wave Doppler image; (a4) severe MR resulting in pulmonary flow reversal; (b1) regurgitant volume; (b2) MR jet area normalized to left atrial area; (b3) pulmonary venous flow systolic to diastolic (S/D) ratio. *p* < 0.05 was considered significant. MR, mitral regurgitation.

Techniques to induce severe MR, were described previously in detail (Corporan et al., 2020, 2018; Corporan, Kono, et al., 2020, 2018; Onohara et al., 2019). Rats were sedated with 5% isoflurane in 100% oxygen for 5 min, intubated, and mechanically ventilated with 2.5% isoflurane in 100% oxygen, with a tidal volume of 1 ml/100 g body weight and a ventilation rate of 66 breaths per minute. The rat was then placed in the right decubitus position for surgery, and pre‐operative antibiotic (Gentamicin, 6 mg/kg, SQ) and analgesic (Carprofen, 2.5 mg/kg, SQ) were administered. The left side of the chest was shaved and the surgical area was scrubbed with Betadine and Ethanol to achieve a sterile surgical field. A skin incision was performed, followed by a left thoracotomy in the fourth intercostal space to access the heart. A pericardiotomy exposed the ventricular apex, and a purse string suture with 6‐0 prolene (8307H, Ethicon) was placed on the apex. An 8Fr transesophageal echocardiographic (TEE) probe (8 MHz, Acunav, Biosense Webster) was inserted into the esophagus to obtain a two‐chamber view of the heart and to measure mitral valve hemodynamics. A saline flushed 23G needle with a stopcock on its proximal end, was inserted through the apical purse string into the LV. The tip of the needle was seen on TEE, and gradually advanced into the anterior mitral leaflet to perforate it (Figure 1a1). Upon puncturing the leaflet, the needle was retracted and severe MR was confirmed with color and spectral Doppler (Figure 1a2). The purse string suture was tightened, the chest was closed in layers with 4‐0 vicryl suture (J496H, Ethicon), and a temporary chest tube (SR‐OX1651CA, Terumo) was inserted to evacuate the thorax of blood and air. Another dose of analgesic (Carprofen, 2.5 mg/kg, SQ) was administered, and the rats were weaned from anesthesia and ventilation, while monitoring SpO_2_ and heart rate. Upon onset of spontaneous breathing, ventilation was ceased, rats were extubated and moved to a clean cage with supplemental oxygen, and warmed with a heating lamp. Longer term pain relief was achieved with Burprenex (0.02 mg/kg, SQ) within 3 h after the rat was ambulatory. For three post‐operative days, rats were administered Carprofen (5 mg/kg, SQ, POD 1–3) and Gentamicin (6 mg/kg, SQ, POD 1–3). Rats were assigned to the experimental groups with different time points for follow‐up. Age‐matched rats were used as control animals and were assigned to groups in a similar manner as the experimental group.

### 2.4 Cardiac echocardiography

At baseline, transthoracic and transesophageal echocardiography were performed in all the rats. In rats induced with MR, imaging was repeated 2 weeks after the surgery and at termination, to confirm the presence of MR and quantify its severity. Transthoracic imaging was performed with a 21 MHz probe on a Visualsonics 2100 ultrasound system (Fujifilm Visualsonics Inc), with the rat sedated and maintained on anesthesia with a nose cone. LV end‐diastolic volumes were measured from parasternal long‐axis B‐mode images using the LV trace tool and calculated using the modified Simpson's monoplane method of disks. All images were analyzed using the Vevo Lab analysis software (v.3.2.0, Fujifilm Visualsonics, Inc.). Transesophageal echo was performed with an 8Fr intracardiac echo probe inserted into the rat's esophagus, while sedated, intubated, and mechanically ventilated using the same ventilation parameters used for surgery. A high esophageal, two‐chamber view of the left atrium and LV were obtained to assess mitral flow and color Doppler for quantification of MR. MR was quantified using three measurements: (1) MR jet area (%) by tracing the regurgitant jet area on color Doppler and normalized to the left atrial area; (2) MR volume (µl) by measuring the MR jet velocity time integral on continuous wave Doppler and multiplying it by the area of the regurgitant orifice created from the 23G needle (0.64 mm OD); (3) pulmonary flow reversal measured by taking the ratio of the pulmonary systolic and diastolic wave velocities.

### 2.5 Whole heart harvest and explant heart decellularization

**FIGURE 2:**
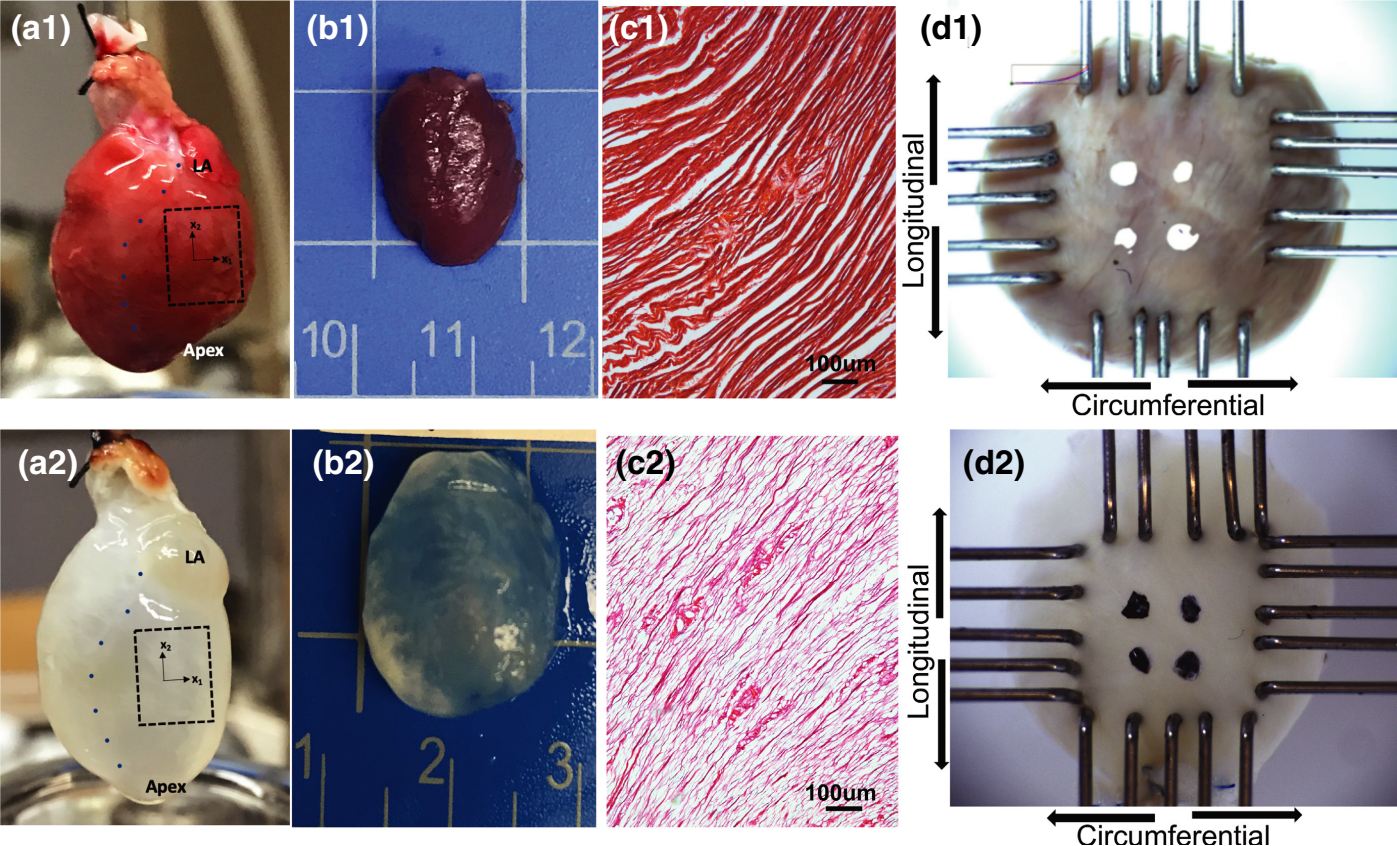
Experimental setup and specimen preparation in intact (a1–d1) and decellularized (a2–d2) hearts. (a1, a2) Whole hearts extracted from rats; (b1, b2) left ventricular free wall cut from the hearts; (c1, c2) hematoxylin and eosin staining of the LV in myocardial region; (d1, d2) LV free wall tissue mounted on the biorakes for equibiaxial tensile testing. LV, left ventricle.

Animals were euthanized with 5% isoflurane in 100% oxygen for 5 min, followed by intracardiac injection of heparin (1cc, McKesson) and then Euthasol (1cc, Virbac Animal Health). Hearts were explanted upon cessation of contractility, and if using intact myocardial sample for mechanical testing, LV free wall was dissected by removing the left atrium and cutting along the interventricular septum from the base to the apex (Figure 2a1–b1,a2–b2). The left ventricular free wall thickness was measured at three distinct locations on each sample using a micrometer (2097A12, McMaster Carr) and averaged. To extract the non‐myocyte myocardial fraction, some hearts were perfusion decellularized (*n* = 5) in each experimental group at each timepoint. Explanted hearts were cannulated through the aorta, and antegrade perfused through the aorta with heparinized 1x phosphate buffered saline (PBS) with 5 mM Adenosine for 15 min, 1% sodium dodecyl sulfate in ultrapure H_2_O (Milli‐Q) for 12–16 h, ultrapure H_2_O for 15 min, and 1% Triton‐X‐100 in ultrapure H_2_O for 20 min (Akhyari et al., 2011).

### 2.6 Characterizing the decellularized myocardium

Decellularization was confirmed with histopathology and scanning electron microscopy (SEM‐ [Topcon DS 130F]) in a subset of rat hearts (intact [*n* = 2] and decellularized [*n* = 2]). For histopathology, explanted hearts were stored in 10% buffered formalin for 24 h, and subsequently transferred to 70% ethanol for long‐term storage. Hearts were embedded in paraffin, and 5 µm thick mid‐ventricular longitudinal sections of intact and decellularized samples were slide‐mounted and stained with hematoxylin and eosin (H & E) and 4′,6‐diamidino‐2‐phenylindole (DAPI) to visualize the presence or absence of cells in intact or decellularized samples, respectively. Samples were also stained with Masson's trichrome to assess the changes that occurred in collagen architecture after decellularization. Histology images were captured with Zeiss AxioScope A1 microscope with a Zeiss AxioCam MRc digital camera. SEM was performed to confirm the presence or lack of cells, as well as to observe the changes in the collagen ultrastructure. For electron microscopy, samples were serially dehydrated, mounted on a metal stub, and were later gold sputter coated with precision etching (Denton desktop) for imaging. To quantitatively validate the decellularization protocol in the study, DNA estimation was performed on all tissue samples using the PureGenome^TM^ tissue DNA extraction kit (Millipore Sigma, 72635) which were later used for mechanical testing.

### 2.7 Biaxial tensile testing

10 × 10 mm LV free wall samples were mounted onto the CellScale® planar biaxial BioTester (CellScale) using custom‐made tines. Samples were mounted such that the baso‐apical axis was aligned in the longitudinal direction (*Y* direction), and the antero‐ posterior axis was aligned in the circumferential direction (*X* direction). White markers were placed in the center of the intact samples, whereas black graphite markers were placed in the center of the decellularized samples (Figure 2d1–d2) to ensure proper contrast for marker tracking. In experiments with intact LV samples, tissues were submerged in modified Krebs‐Henseleit buffer without calcium, (118 mM NaCl, 4.7 mM Kcl, 1.2 mM MgSO_4_, 1.2 mM KH_2_PO_4_, 10 mM glucose, 25 mM NaHCO_3_, 0.5 mM EDTA, 2 mM sodium pyruvate, 10 mM 2,3‐butanedione monoxime) and warmed to 37 °C to maintain a physiological environment and inhibit cardiomyocyte contraction. Biaxial testing of decellularized LV samples were performed in 1X PBS solution maintained at 37°C, since the extracellular matrix does not require nutrients for cellular activity. Each sample was then subjected to a preload of 10 mN in the circumferential (*X*) and longitudinal (*Y*) loading directions, followed by six pre‐conditioning cycles of equibiaxial 30% strain to remove hysteresis. Four additional cycles of equibiaxial 30% strain cycles were performed, and axis displacement, axis force, and marker displacement data from these cycles were obtained, averaged, and analyzed. The range of strains were obtained from *in vivo* measurements on myocardium reported previously (Ishizu et al., 2014; Kocabay et al., 2014; Narayan et al., 2017). The nominal stress tensor was calculated from the force measured by the load cells (10 N) during each strain cycle, divided by the cross‐sectional area of the sample measured at the beginning of the experiment, defined as *sample length* *×* *thickness*. Engineering strain **E** was calculated by tracking the displacement of the optical markers, using the LabJoy® software (CellScale). Stress‐strain data was fit to a simplified exponential model (Demer & Yin, 1983):

$$\text{Stress}=a\times e^{b(\text{strain})}+c,$$

and material parameters (*a*, *b*, *c*) were estimated using least‐squares regression analysis. To compute the elastic modulus for each sample, the slope in the linear region of the stress‐strain curve was calculated for each sample. Extensibility, a mechanical property which describes the extent to which the material can be stretched, was determined at low (5 kPa), mid (1/2 of the maximum stress), and high (maximum stress) region for each sample by measuring the corresponding strain values. Anisotropy index was calculated as a ratio of the maximum strain in the circumferential to the longitudinal direction.

### 2.8 Statistical analysis

Data analysis and statistics were performed in Prism GraphPad 7.0 (GraphPad Software Inc). All data were tested for normality using the D’Agostino & Pearson or Shapiro–Wilk test. Data following a gaussian distribution is represented as mean ± 1 standard deviation and non‐normally distributed data is represented as median and interquartile range (IQR; 25%–75%). MR severity at baseline and 2 weeks were compared using a paired *t*‐test for normally distributed data and Wilcoxon matched‐pairs test for and non‐parametric data. For cardiac parameters and material properties, normally distributed data was analyzed with a one‐way ANOVA with Tukey's multiple comparisons test while non‐parametric data was analyzed with a Mann Whitney test with Dunn's correction and multiple comparisons. *p* < 0.05 was considered statistically significant.

## 3 RESULTS

### 3.1 Quantification of MR

Survival after the surgery was 100%. MR was confirmed in all rats after the surgery, using color Doppler and pulsed‐wave Doppler imaging (Figure 1a1–a4). At 2 weeks after surgery to induce MR, regurgitant volume was 126.3 ± 32.1 µl/beat compared to 0 µl/beat at baseline (*p* < 0.0001) (Figure 1b1). MR jet area normalized to the left atrial area was 40.4% (IQR: 35.1–44.6) compared to 0% at baseline (*p* < 0.0001) (Figure 1b2). Pulmonary venous flow reversal was observed with a change in the systolic to diastolic velocity (S/D) ratio from 0.76 ± 0.19 at baseline to −0.70 ± 0.99 at 2 weeks (*p* < 0.0001) (Figure 1b3).

### 3.2 Left ventricular morphological changes

Changes in left ventricular morphology are summarized in Table 1. The percent difference in the end‐diastolic volume between the control and MR groups at 2 weeks was 15.0% (*p* = 0.43), at 10 weeks was 25.5% (*p* = 0.031), at 20 weeks was 23.5% (*p* > 0.99), and 40 weeks was 43.5% (*p* = 0.048). End‐systolic volumes in the MR groups were increased by 2.3% at 2 weeks (*p* > 0.99), 40.5% at 10 weeks (*p* = 0.12), 63.0% at 20 weeks (*p* < 0.0001), and 81.6% at 40 weeks (*p* < 0.0001) compared with control. Ejection fraction was increased at 2 weeks compared to control (71.3 ± 2.5 vs. 67.7 ± 1.1, *p* = 0.09) and was significantly lower at 20 and 40 weeks (*p* < 0.0001). LVEDd was larger in the MR group compared to control at all time‐points. By 40 weeks, LVEDd was significantly higher in the MR group compared to control (9.5 ± 1.0 mm vs. 8.2 ± 0.3 mm, *p* = 0.027). As left ventricular chamber volume increased over the 40 weeks, heart weight and left ventricular mass also increased compared to control. By 10 weeks, LV mass was significantly higher compared to control (1970.6 ± 355.2 vs. 1336.0 ± 229.7 mg, *p* = 0.0003). At 20 weeks, ventricular wall thickness was significantly higher compared to control (*p* = 0.033), which was not the case in the other groups at any of the time points.

**TABLE 1:** Passive cardiac parameters are measured by echocardiography and tissue measurements.

	2 weeks			10 weeks			20 weeks			40 weeks		
	Control	MR	*p*‐value	Control	MR	*p*‐value	Control	MR	*p*‐value	Control	MR	*p*‐value
LVEDd (mm)	7.9 ± 0.2	8.6 ± 0.6	0.73	7.73 ± 0.41	8.54 ± 1.5	0.35	8.2 ± 0.4	8.9 ± 0.9	0.10	8.2 ± 0.3	9.5 ± 1.0	0.027
LVEDV (µl)	527.6 ± 19.9	606.7 ± 58.0	0.43	539.8 ± 40.8	677.2 ± 122.6	0.031	628.2 ± 39.5	776.0 ± 215.9	>0.99	603.5 ± 29.0	866.0 ± 156.0	0.048
LVESV (µl)	170.6 ± 11.6	174.6 ± 27.5	>0.99	170.0 ± 11.3	238.9 ± 47.7	0.12	205.8 ± 24.7	335.5 ± 116.1	<0.0001	207.8 ± 16.0	377.3 ± 85.2	<0.0001
SV (µl)	357.0 ± 10.0	432.1 ± 36.6	0.0017	369.8 ± 32.7	438.3 ± 85.7	0.35	422.4 ± 23.9	440.5 ± 107.2	>0.99	395.7 ± 16.1	488.7 ± 76.3	0.41
EF (%)	67.7 ± 1.1	71.3 ± 2.5	0.09	68.5 ± 1.4	64.9 ± 4.0	0.09	67.3 ± 2.4	57.3 ± 4.4	<0.0001	65.6 ± 1.4	56.7 ± 3.2	<0.0001
Heart weight (g)	1.3 ± 0.1	1.5 ± 0.1	0.30	1.4 ± 0.1	1.6 ± 0.2	0.61	1.6 ± 0.1	1.8 ± 0.3	>0.99	1.6 ± 0.1	1.9 ± 0.3	>0.99
LV mass (mg)	1275.6 ± 165.3	1577.3 ± 253.2	0.35	1336.0 ± 229.7	1970.6 ± 355.2	0.0003	1367.4 ± 155.1	1450.6 ± 483.5	0.99	1525.0 ± 158.6	1860.5 ± 427.0	0.22
Wall thickness (mm)	3.1 ± 0.1	3.2 ± 0.2	>0.99	3.2 ± 0.1	3.1 ± 0.3	>0.99	3.5 ± 0.2	3.0 ± 0.1	0.033	3.3 ± 0.1	3.1 ± 0.3	>0.99

Note

Data are represented as mean ± standard deviation.

### 3.3 Whole heart decellularization

**FIGURE 3:**
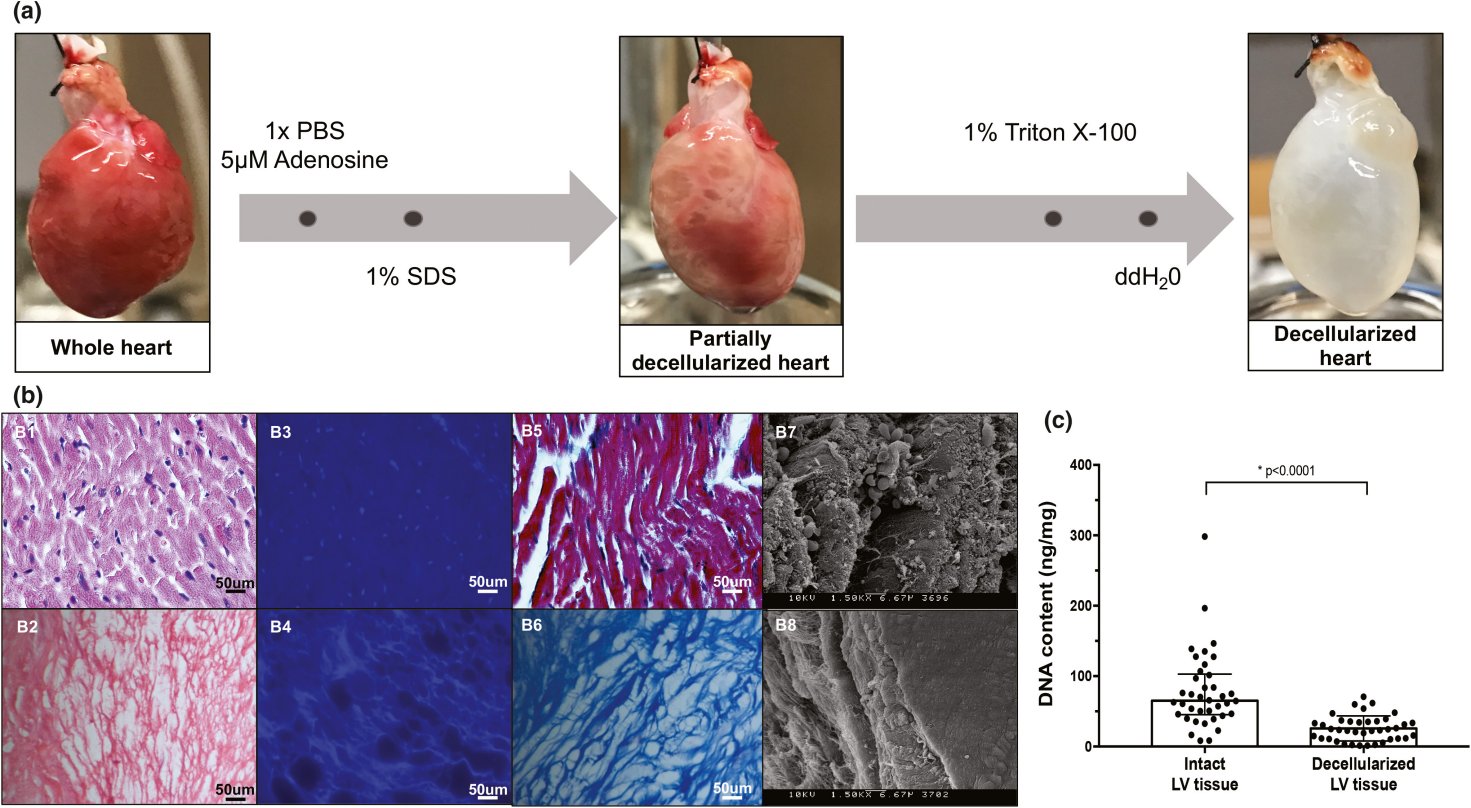
(a) Schematic of the whole heart decellularization process. (b, c) Validation of decellularization procedure. (b1, b2) Hematoxylin and eosin staining; (b3, b4) DAPI staining; (b5, b6) Trichrome staining; and (b7, b8) scanning electron microscopy images of intact and decellularized LV samples. (c) DNA content measured from all intact and decellularized LV samples used for analysis. Normally distributed data is represented as mean ± one standard deviation, and nonparametric data is represented as median with interquartile range. *p* ≤ 0.05 is considered statistically significant. DAPI, 4′,6‐diamidino‐2‐phenylindole.

**FIGURE 4:**
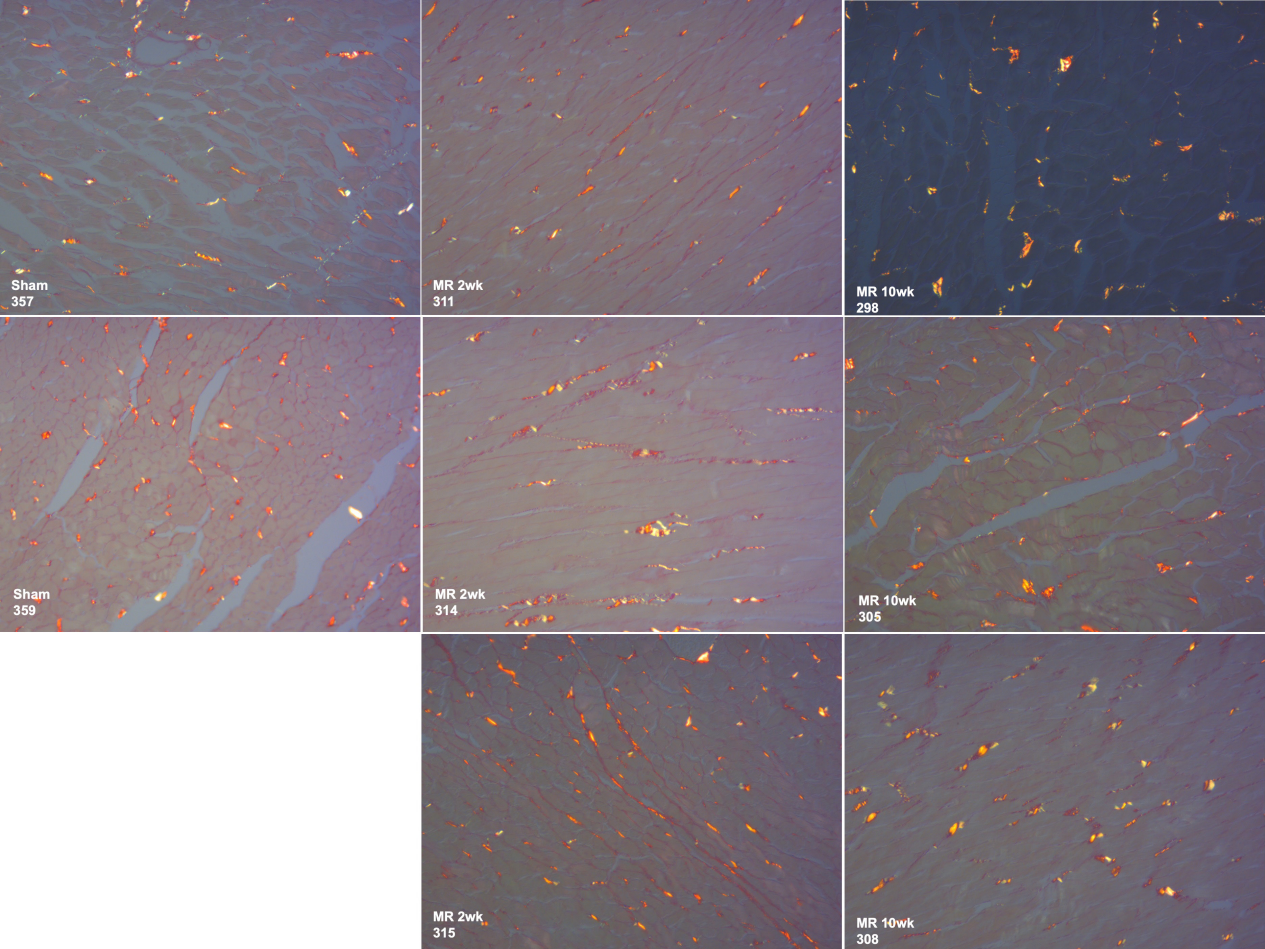
Picrosirius red staining of the left ventricular myocardium for interstitial and perivascular collagen. There were no differences in collagen staining between the groups.

Decellularization of the intact heart resulted in a translucent LV scaffold that was grossly intact compared to the native heart (Figure 3a). H & E staining shown in Figure 3b1–b2, depicts removal of cellular components after decellularization, and a tissue with intact extracellular matrix proteins. Nuclear specific DAPI staining further confirmed cell removal, shown in Figure 3b3–b4. DNA quantification demonstrated a 67.37% reduction in the cellular content after decellularization (*p* < 0.0001 compared to intact tissue), with remnant DNA likely from small DNA fragments left behind from decellularization (Figure 3c). Masson's trichrome staining depicts a contiguous collagen fibril network with pockets where cells were removed, as shown in Figure 3b5–b6. Electron microscopy confirmed the structural integrity of the extracellular compartment of the decellularized scaffolds at high magnification, as shown in Figure 3b7–b8. Additional staining with Picrosirius red was performed to illustrate the changes, if any, in the pericellular collagen content between the groups, shown in Figure 4. There were no differences in the staining between the groups.

### 3.4 Material properties of intact myocardium in MR versus control

**FIGURE 5:**
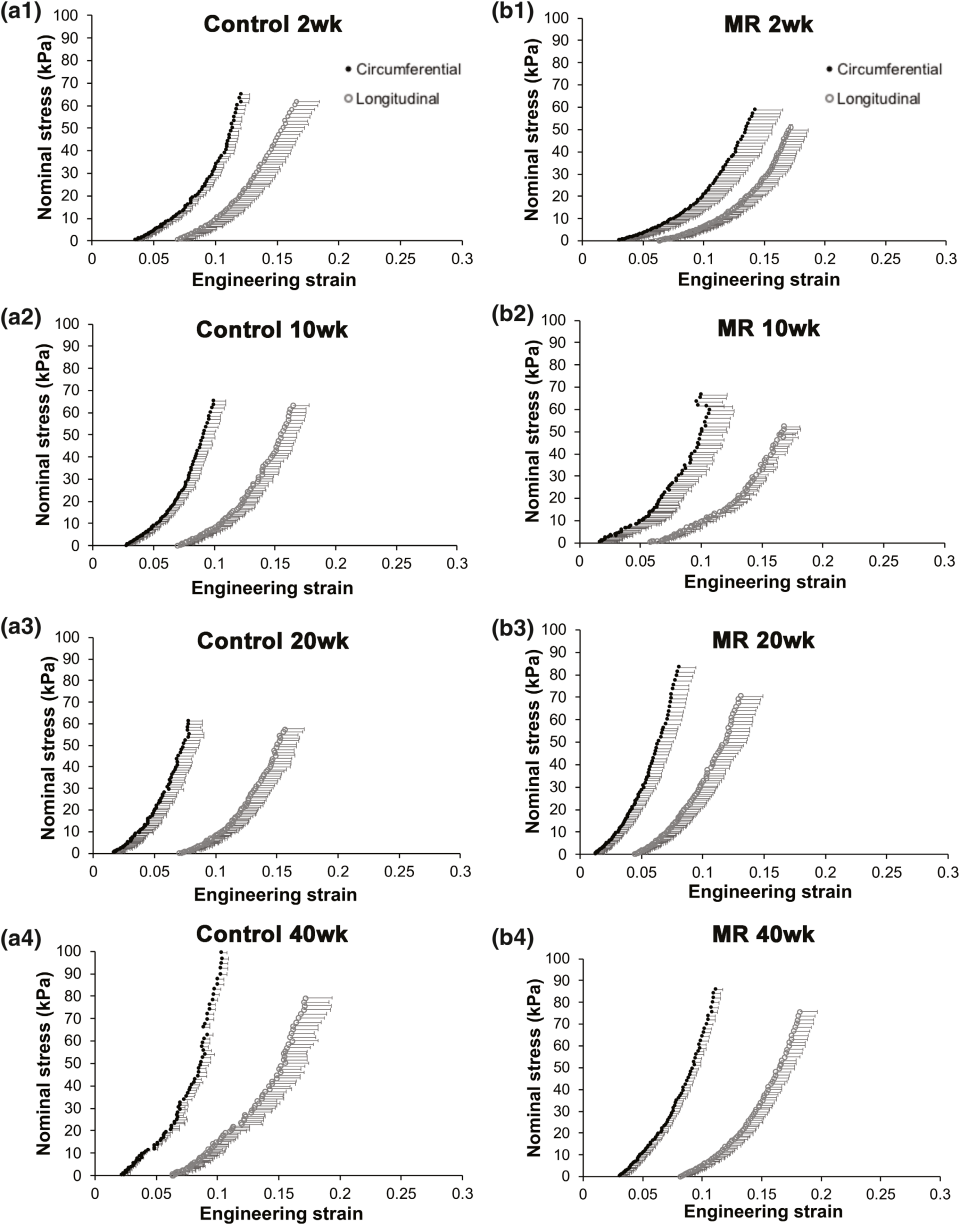
Average stress strain relationships of intact left ventricular tissue in the control (a) and MR (b) groups at 2, 10, 20, and 40 weeks. The circumferential direction is represented as filled black circles and the longitudinal direction is represented as unfilled circles. Error bars represent the standard error. MR, mitral regurgitation.

**FIGURE 6:**
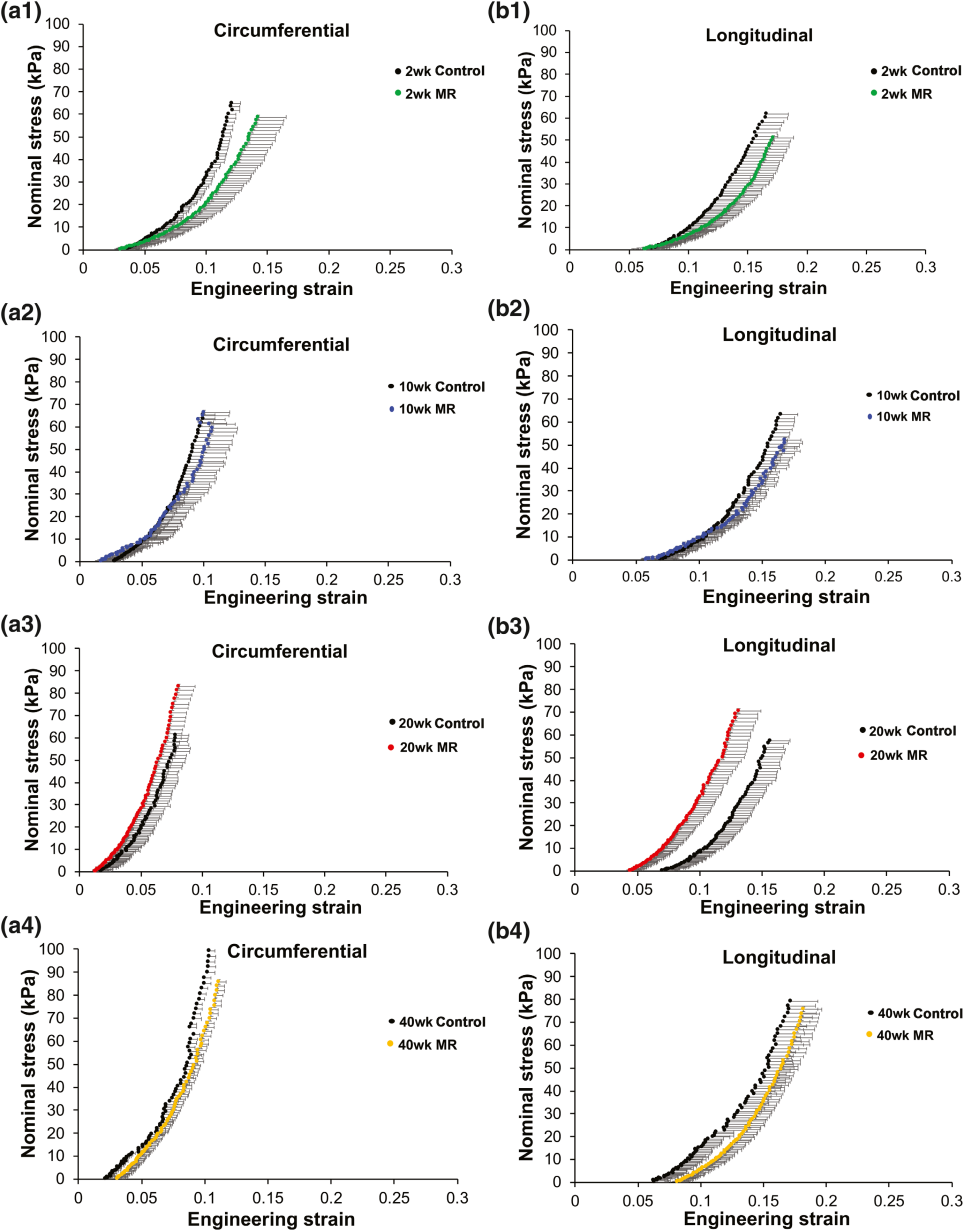
Average stress strain relationships in the circumferential (a) and longitudinal (b) direction in control and MR intact left ventricular tissue. Black data points represent the control groups and the colored data points represent the MR groups at each time‐point. Error bars represent standard error. MR, mitral regurgitation.

**FIGURE 7:**
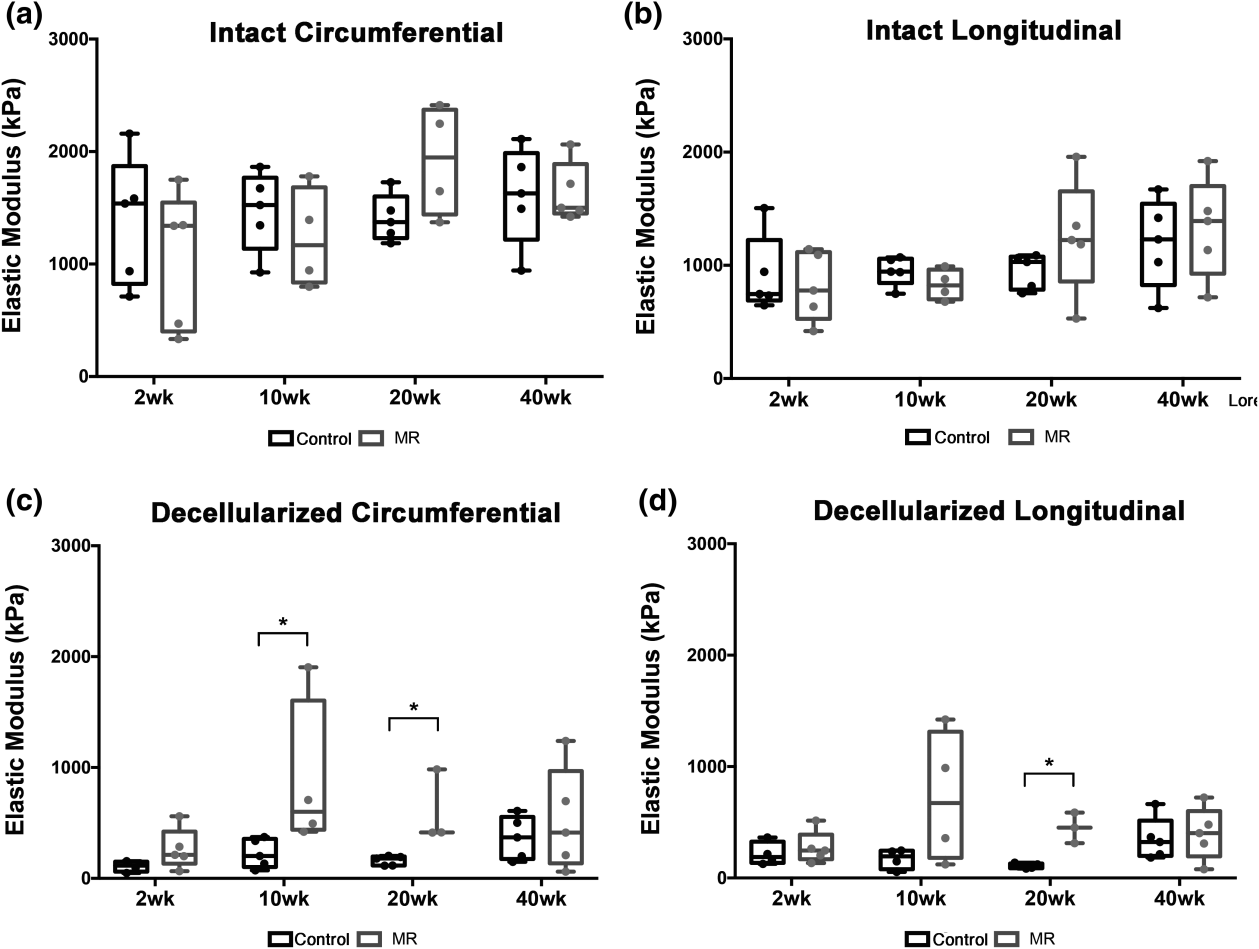
The median passive stiffness of (a) the intact myocardium in the circumferential direction; (b) intact myocardium in the longitudinal direction; (c) in the decellularized myocardium in the circumferential direction, and (d) decellularized myocardium in the longitudinal direction at 2, 10, 20, and 40 weeks. Bar graphs represent median and interquartile. *p* < 0.05 is considered statistically significant, and denoted with a *.

Stress‐strain relationships for intact LV tissue are shown in Figure 5. Material properties, such as extensibility and mechanical coefficients derived from the stress‐strain plots, are summarized in Tables 2 and 3. In both the control and MR groups, intact LV tissue exhibited a stiffer response in the circumferential loading direction compared to the longitudinal direction at 2, 10, 20, and 40 weeks, indicating anisotropy. In the control group, intact LV tissue exhibited a high degree of anisotropy at all time‐points (0.56–0.72) (Table 2). In the MR 2 week group, intact LV tissue exhibited an anisotropy index of 0.79 (IQR: 0.51–1.30), indicating the lowest degree of anisotropy. By 10, 20, and 40 weeks, the anisotropy index was higher (0.44–0.63) in the intact LV tissue. Extensibility of the intact tissue in the MR group at different regions of stress (high, mid, and low) was the lowest in the 20‐week group in both the circumferential and longitudinal directions. In the circumferential direction, the material coefficient (a) which corresponds to the slope of the high strain region was the highest in the MR 40‐week group with a median of 16.03 (Table 3). The material coefficient (b), which corresponds to chamber stiffness from the linear elastic range, was highest at 20 weeks in the circumferential direction. In the longitudinal direction, the MR group had the highest material coefficients at 20 weeks. Figure 6 shows the average stress‐strain relationships comparing control and MR groups at each time‐point in intact tissue. At 2 weeks, the LV intact tissue exhibited a rightward shift, in both the circumferential and longitudinal directions in the MR group (green) compared to control. At 10 weeks, the stress‐strain curves in the MR group (blue) did not differ in the circumferential and longitudinal directions compared to control. By 20 weeks, the MR group (red) showed a leftward shift in the curves, which was pronounced in the longitudinal direction compared to control. Finally, by 40 weeks, the stress‐strain curves in the MR group (yellow) exhibited a rightward shift in the longitudinal direction compared to control but did not differ in the circumferential direction. The median elastic moduli of the intact myocardium in control and MR tissues are shown in Figure 7a,b. In the control group, the elastic modulus of the intact tissue did not differ at 2, 10, 20, and 40 weeks in both the circumferential and longitudinal directions. The median elastic modulus of the control group increased after 40 weeks. After 2 weeks of MR, the elastic modulus in the circumferential decreased compared to control (1341 kPa [IQR: 835.7–1683] vs. 1539 kPa [IQR: 824.2–1871], *p* = 0.99), although it did not reach statistical significance. Stiffness in the longitudinal direction was similar to the control tissue at 2 weeks that did not show any variation. There were no changes observed in the elastic modulus of the intact tissue after 10 weeks of MR compared to control, however, the stiffness increased by 20 weeks. In the circumferential direction, the elastic modulus also increased compared to control (1948 kPa [IQR: 1441–2372] vs. 1373 kPa [IQR: 1231–1602], *p* = 0.19). In the longitudinal direction, the elastic modulus was also increased compared to control (1224 kPa [IQR: 857.8–1654] vs. 1029 kPa [IQR: 785–1076], *p* = 0.15). By 40 weeks in the MR group, the elastic modulus was 1500 kPa (IQR: 1450–1889) in the circumferential direction and was 1391 kPa (IQR: 926.4–1700) in the longitudinal direction. In general, the elastic modulus calculated from the stress‐strain curves increased from 2 to 40 weeks.

**TABLE 2:** Indices of passive material properties of intact LV tissue.

	2 weeks		10 weeks		20 weeks		40 weeks	
	Control	MR	Control	MR	Control	MR	Control	MR
Anisotropy index	0.72 (0.65–0.91)	0.79 (0.51–1.30)	0.61 (0.56–0.65)	0.63 (0.49–0.86)	0.56 (0.40–0.61)	0.44 (0.42–0.93)	0.59 (0.48–0.81)	0.63 (0.51–0.75)
*E*_max, circ_	0.13 (0.11–0.13)	0.11 (0.10–0.19)	0.10 (0.08–0.12)	0.11 (0.07–0.15)	0.08 (0.06–0.10)	0.07 (0.06–0.11)	0.10 (0.09–0.11)	0.12 (0.10–0.12)
*E*_max, long_	0.18 (0.12–0.20)	0.15 (0.14–0.21)	0.17 (0.13–0.19)	0.16 (0.15–0.19)	0.17 (0.12–0.18)	0.14 (0.09–0.16)	0.16 (0.13–0.22)	0.19 (0.15–0.21)
*E*_mid, circ_	0.10 (0.09–0.11)	0.09 (0.08–0.16)	0.08 (0.06–0.10)	0.09 (0.06–0.12)	0.06 (0.04–0.08)	0.05 (0.04–0.08)	0.08 (0.07–0.09)	0.09 (0.08–0.10)
*E*_mid, long_	0.15 (0.10–0.16)	0.14 (0.11–0.18)	0.14 (0.11–0.16)	0.13 (0.13–0.15)	0.14 (0.10–0.16)	0.11 (0.07–0.13)	0.13 (0.10–0.19)	0.16 (0.13–0.17)
*E*_low, circ_	0.05 (0.04–0.06)	0.04 (0.03–0.10)	0.04 (0.03–0.05)	0.04 (0.01–0.07)	0.03 (0.01–0.05)	0.02 (0.01–0.03)	0.03 (0.02–0.04)	0.04 (0.03–0.05)
*E*_low, long_	0.10 (0.06–0.11)	0.11 (0.08–0.44)	0.10 (0.07–0.11)	0.08 (0.07–0.10)	0.10 (0.06–0.11)	0.06 (0.04–0.08)	0.07 (0.05–0.11)	0.11 (0.08–0.12)

Note

Data are represented as median and interquartile range (IQR; 25%–75%).

Abbreviations: LV, left ventricle; MR, mitral regurgitation.

**TABLE 3:** Coefficients for exponential stress‐strain relationships for intact tissue.

	Circumferential			Longitudinal		
	*a*	*b*	*c*	*a*	*b*	*c*
2 weeks						
Control	4.19 (3.07–5.05)	23.84 (19.69–27.40)	−9.01 (−11.39 to −8.83)	2.82 (2.22–10.27)	17.68 (16.08–20.74)	−11.31 (−22.36 to −8.59)
MR	5.50 (2.02–7.80)	21.46 (15.85–24.42)	−9.85 (−10.9 to −4.22)	1.98 (0.46–2.98)	20.37 (18.03–30.61)	−3.95 (−9.43 to −1.81)
10 weeks						
Control	4.73 (3.07–5.42)	29.18 (24.05–36.12)	−9.85 (−11.39 to −7.31)	1.76 (1.12–4.17)	21.44 (19.93–24.16)	−8.35 (−11.13 to −7.52)
MR	4.19 (3.73–13.68)	20.22 (18.85–34.19)	−9.28 (−11.6 to −4.96)	2.51 (2.07–2.75)	19.15 (16.52–21.88)	−6.78 (−8.73 to −6.61)
20 weeks						
Control	7.24 (4.09–56.65)	23.68 (15.48–29.91)	−14.80 (−58.02 to −9.71)	1.49 (0.98–2.22)	22.81 (21.35–30.01)	−8.21 (−10.14 to −6.09)
MR	13.29 (12.62–16.14)	28.80 (17.79–34.56)	−19.91 (−20.98 to −17.72)	4.74 (3.78–9.13)	20.12 (17.13–29.39)	−14.22 (−16.79 to −11.26)
40 weeks						
Control	12.95 (10.87–19.01)	19.60 (14.92–23.48)	−20.83 (−26.71 to −18.4)	4.10 (2.71–7.99)	17.80 (13.09–24.64)	−12.14 (−14.9 to −11.03)
MR	16.03 (6.69–23.75)	18.16 (14.60–26.43)	−25.51 (−33.72 to −14.97)	1.90 (1.41–5.86)	19.52 (15.59–25.18)	−11.93 (−18.74 to −7.99)

Note

Data are represented as median and interquartile range (IQR; 25%–75%).

Abbreviation: MR, mitral regurgitation.

### 3.5 Material properties of decellularized myocardium in MR versus control

**FIGURE 8:**
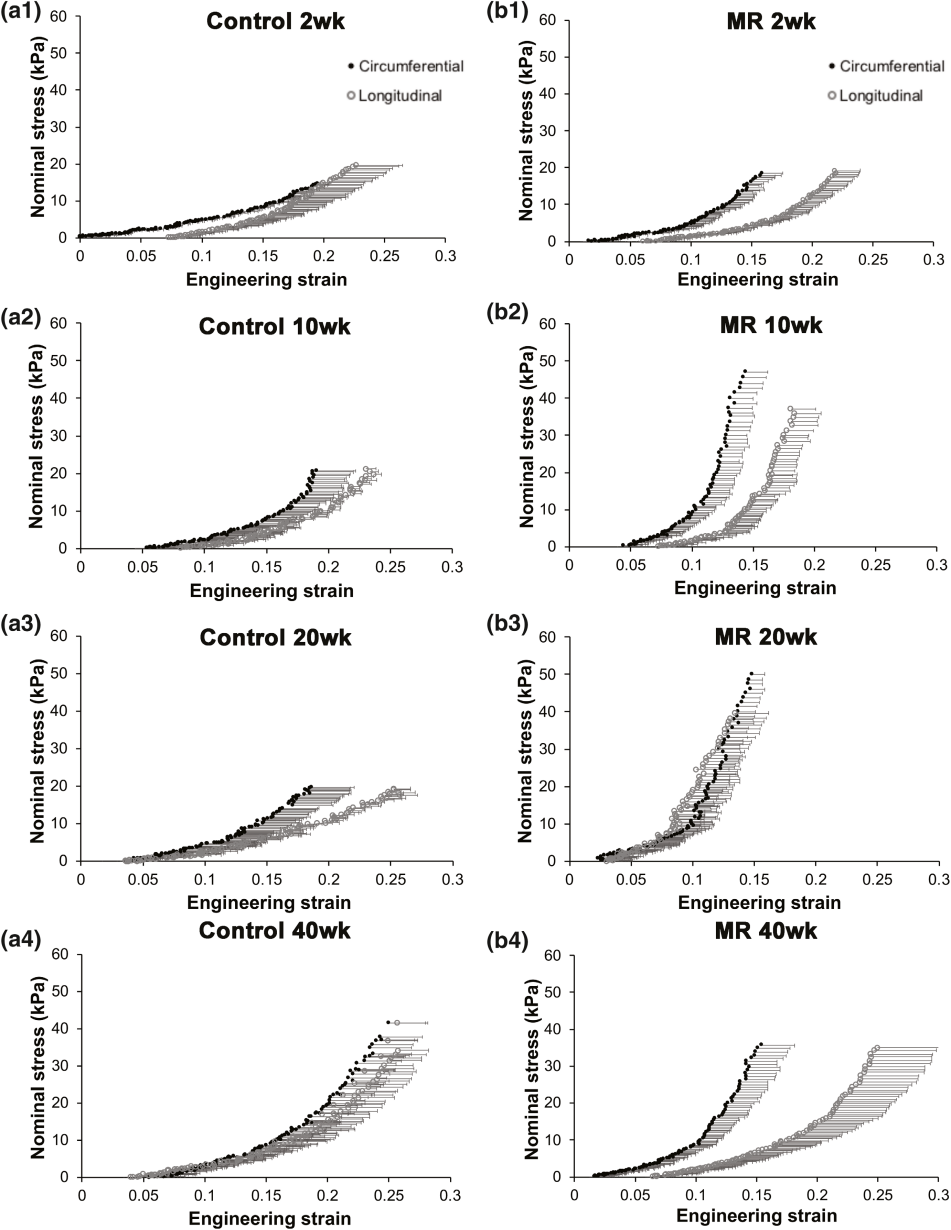
Average stress strain relationships of decellularized left ventricular tissue in the control (a) and MR (b) groups at 2, 10, 20, and 40 weeks. The circumferential direction is represented as filled black circles and the longitudinal direction is represented as unfilled circles. Error bars represent the standard error. MR, mitral regurgitation.

**FIGURE 9:**
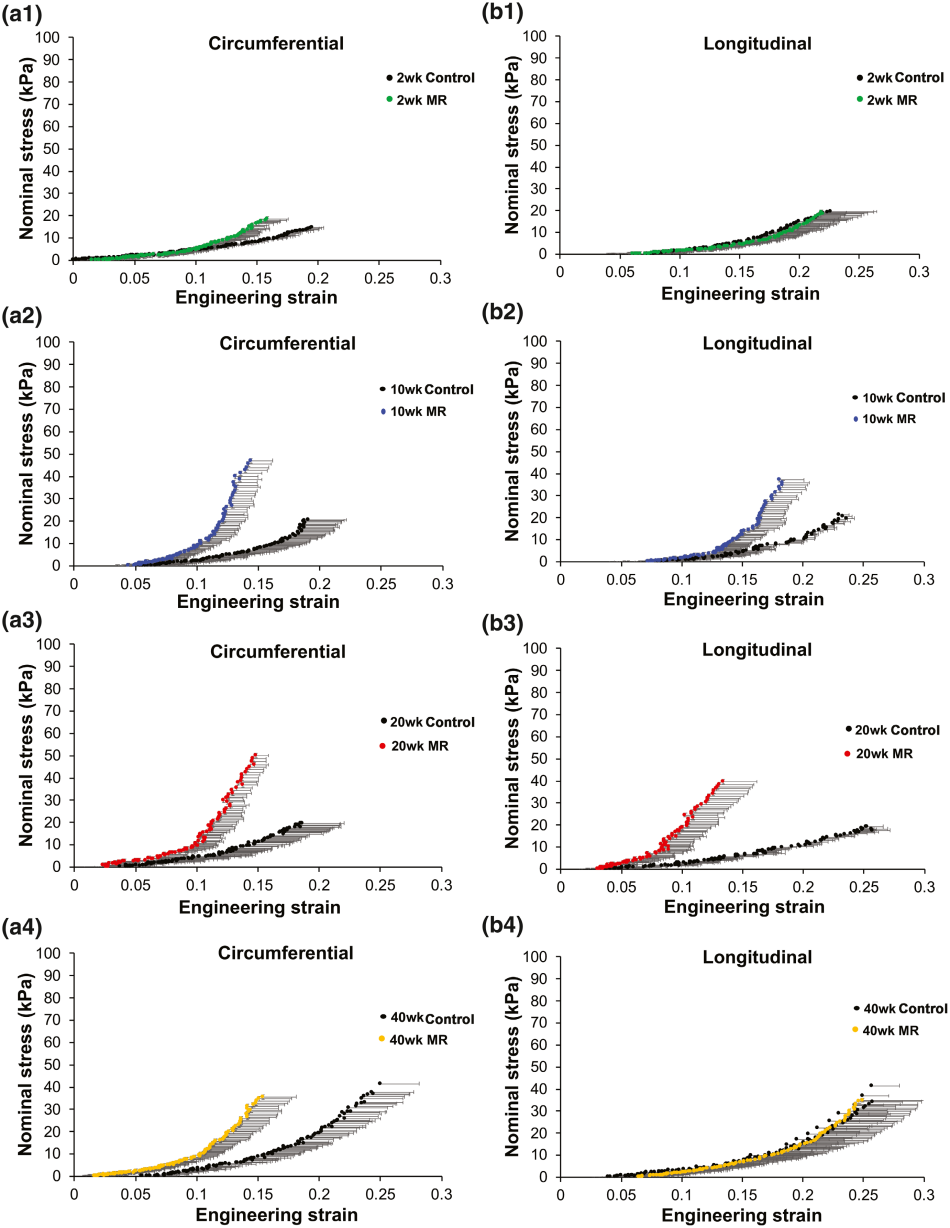
Average stress strain relationships in the circumferential (a) and longitudinal (b) direction in control and MR decellularized left ventricular tissue. Black data points represent the control groups and the colored data points represent the MR groups at each time‐point. Error bars represent standard error. MR, mitral regurgitation.

Stress‐strain plots for decellularized LV tissue are shown in Figure 8. Material properties derived from the stress‐strain data, are summarized in Tables 4 and 5. In the control group, the decellularized tissue exhibited a lesser anisotropic response, compared to intact LV tissue, with anisotropy index values ranging between 0.74 and 1.10 (Figure 8, Table 4). The MR group exhibited a higher degree of anisotropy (stiffer circumferential response) at 2, 10, and 40 weeks (Table 4). By 20 weeks, the median anisotropy index was 0.94. In the MR group, maximum extensibility in the circumferential direction was the lowest at 10 weeks, mid extensibility was the lowest at 2 and 10 weeks, and low extensibility was the lowest at 40 weeks. In the longitudinal direction, maximum, mid, and low extensibility was the lowest at 20 weeks in the MR group. Figure 9 shows the average stress‐strain relationships directly comparing control and MR groups at each time‐point in decellularized tissue. In the decellularized tissue, the stress‐strain relationships showed a different trend throughout the time‐points compared to intact tissue. At 2 weeks, the LV decellularized tissue exhibited a leftward shift of the stress‐strain curve only in the circumferential direction in the MR group (green) compared to control, primarily in the higher strain region. At 10 weeks, the stress‐strain curves in the MR group (blue) exhibited a leftward shift in both the circumferential and longitudinal directions compared to control. By 20 weeks, the MR group (red) maintained the leftward shift in the curves in the circumferential and longitudinal directions compared to control. By 40 weeks, the stress‐strain curves in the MR group (yellow) exhibited a leftward shift only in the circumferential direction compared to control. The median elastic moduli of the decellularized myocardium in control and MR tissues are shown in Figure 7c,d. For the decellularized LV tissues in the control group, the elastic modulus was unchanged at 2, 10, and 20 weeks in both the circumferential and longitudinal directions. By 40 weeks, the elastic modulus increased in both the circumferential and longitudinal directions. In the MR group, the elastic modulus increased in the circumferential direction at 2 weeks compared to control (212.1 kPa [IQR: 132.4–422.9] vs. 115.1 kPa [IQR: 60.52–150.4], *p* = 0.11). By 10 weeks, the elastic modulus was increased compared to control in the circumferential direction (600.4 kPa [IQR: 439–1605] vs. 201.8 kPa [IQR: 102.5–355.6], *p* = 0.016) and in the longitudinal direction (672.3 kPa [IQR: 180.7–1314] vs. 104.6 kPa [IQR: 88.49–129.7], *p* = 0.20). By 20 weeks, the elastic modulus remained significantly higher in both the circumferential and longitudinal directions, compared to control. In the circumferential direction, the elastic modulus was 415.4 kPa (IQR: 413.1–983.7) compared to control (179.1 kPa [IQR: 116–197.6], *p* = 0.036). In the longitudinal direction, the elastic modulus was 452.4 kPa (IQR: 312.4–586.9) compared to control (104.6 kPa [IQR: 88.49–129.7], *p* = 0.036). By 40 weeks, there were no differences in the elastic modulus compared to control.

**TABLE 4:** Indices of passive material properties of decellularized LV tissue.

	2 weeks		10 weeks		20 weeks		40 weeks	
	Control	MR	Control	MR	Control	MR	Control	MR
Anisotropy index	0.97 (0.63–1.30)	0.63 (0.56–1.05)	0.87 (0.50–1.24)	0.67 (0.54–1.53)	0.74 (0.47–0.90)	0.94 (0.89–1.99)	1.10 (0.76–1.17)	0.64 (0.43–0.96)
*E*_max, circ_	0.19 (0.18–0.22)	0.14 (0.13–0.20)	0.17 (.13–0.26)	0.13 (0.11–0.19)	0.22 (0.11–0.23)	0.14 (0.13–0.17)	0.24 (0.19–0.32)	0.14 (0.10–0.22)
*E*_max, long_	0.22 (0.15–0.31)	0.23 (0.17–0.26)	0.22 (0.21–0.25)	0.19 (0.13–0.22)	0.25 (0.23–0.28)	0.14 (0.07–0.19)	0.26 (0.21–0.30)	0.21 (0.16–0.36)
*E*_mid, circ_	0.13 (0.11–0.14)	0.12 (0.10–0.16)	0.14 (0.11–0.22)	0.12 (0.08–0.17)	0.16 (0.08–0.19)	0.13 (0.08–0.16)	0.21 (0.14–0.25)	0.14 (0.08–0.15)
*E*_mid, long_	0.17 (0.12–0.23)	0.20 (0.15–0.21)	0.20 (0.18–0.21)	0.17 (0.11–0.21)	0.20 (0.16–0.22)	0.08 (0.06–0.17)	0.21 (0.16–0.25)	0.18 (0.13–0.31)
*E*_low, circ_	0.04 (0.02–0.07)	0.06 (0.04–0.11)	0.09 (0.06–0.18)	0.12 (0.09–0.16)	0.08 (0.03–0.09)	0.08 (0.001–0.09)	0.09 (0.07–0.13)	0.06 (0.03–0.08)
*E*_low, long_	0.10 (0.08–0.13)	0.12 (0.09–0.14)	0.11 (0.10–0.13)	0.17 (0.11–0.21)	0.08 (0.06–0.12)	0.06 (0.002–0.11)	0.09 (0.05–0.14)	0.08 (0.05–0.19)

Note

Data are represented as median and interquartile range (IQR; 25%–75%).

Abbreviations: LV, left ventricle; MR, mitral regurgitation.

**TABLE 5:** Coefficients for exponential stress–strain relationships for decellularized tissue.

	Circumferential			Longitudinal		
	*a*	*b*	*c*	*a*	*b*	*c*
2 weeks						
Control	2.40 (1.22–11.64)	9.06 (4.63–15.09)	−2.17 (−12.43 to −0.37)	2.71 (0.79–3.48)	10.86 (6.91–26.52)	−5.31 (−6.84 to −2.60)
MR	0.73 (0.16–3.85)	21.22 (12.58–29.27)	−2.25 (−4.06 to −0.35)	0.30 (0.04–2.90)	21.16 (9.93–32.42)	−1.66 (−4.27 to −0.70)
10 weeks						
Control	1.46 (0.30–4.79)	14.71 (8.26–22.54)	−3.25 (−6.19 to −0.90)	0.77 (0.32–2.22)	15.57 (8.15–19.26)	−2.45 (−3.66 to −1.28)
MR	0.28 (0.23–2.27)	31.13 (22.99–41.37)	−3.10 (−4.71 to −1.61)	0.30 (0.09–0.48)	26.92 (20.76–39.18)	−2.60 (−3.48 to −1.09)
20 weeks						
Control	2.59 (1.19–3.48)	11.84 (9.34–19.67)	−2.99 (−3.43 to −2.13)	2.34 (1.82–14.16)	8.79 (4.98–9.45)	−3.20 (−14.93 to −3.05)
MR	0.06 (0.05–25.73)	34.69 (13.03–42.83)	−0.73 (−27.65 to −0.29)	0.23 (0.09–62.22)	25.48 (5.84–67.45)	−1.91 (−61.42 to −0.94)
40 weeks						
Control	1.43 (0.93–7.65)	11.53 (7.37–20.49)	−4.18 (−9.86 to −3.06)	1.37 (0.59–18.93)	12.78 (7.10–19.47)	−3.25 (−20.1 to −1.10)
MR	3.34 (1.56–4.46)	20.29 (8.88–26.78)	−4.48 (−6.36 to −2.33)	1.19 (0.67–3.67)	13.68 (10.04–18.94)	−3.33 (−5.05 to −2.84)

Note

Data are represented as the median and interquartile range (IQR; 25%–75%).

Abbreviation: MR, mitral regurgitation.

### 3.6 Contribution of the extracellular matrix to overall material properties of the LV

On average, the elastic modulus of decellularized LV tissue was lower than the elastic modulus for intact LV tissue. At 2 weeks, the decellularized tissue only contributed up to 25.2% and 33.52% to the overall elastic modulus of the intact tissue in the circumferential and longitudinal directions, respectively. At 10 weeks, the modulus for the intact LV was not significantly changed, however, the decellularized tissue was stiffest in both directions. At 10 weeks, the elastic modulus of the decellularized tissue contributed up to 71.72% and 88.17% to the overall elastic modulus of the intact tissue in the circumferential and longitudinal directions, respectively. The elastic modulus was highest at 20 weeks for intact tissue in the circumferential direction, and the decellularized tissue contributed up to 31.46% and 36.08% to the overall change in the elastic modulus of the intact tissue in the circumferential and longitudinal directions, respectively. At the 40‐week time point, the elastic modulus in the intact tissue was highest in the longitudinal direction and the decellularized tissue only contributed up to 32.06% and 29.98% to the overall elastic modulus of the intact tissue in the circumferential and longitudinal directions, respectively.

### 3.7 Correlation of left ventricular passive mechanics and geometry

**FIGURE 10:**
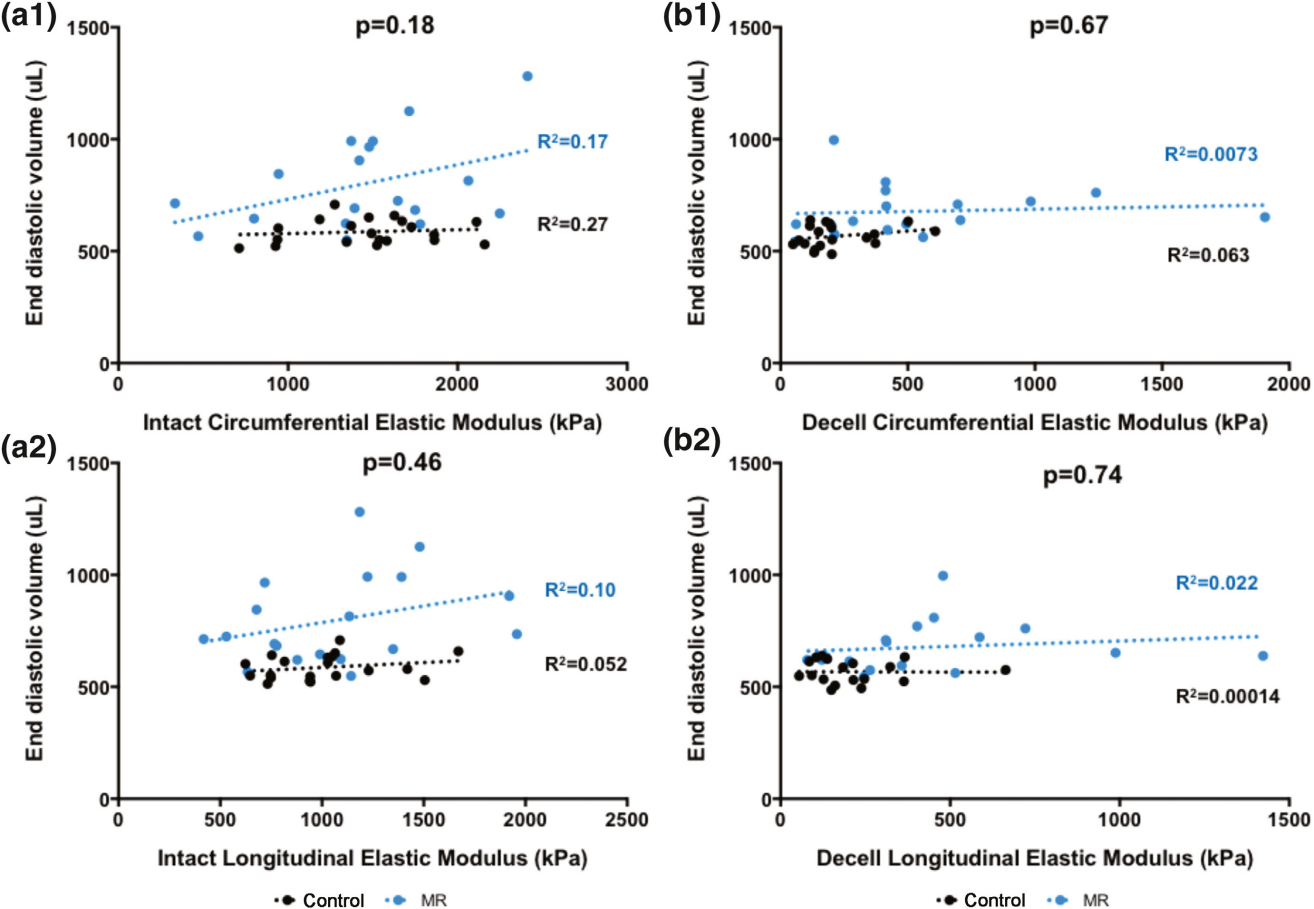
(a1) Intact circumferential elastic modulus and (a2) intact longitudinal elastic modulus versus end‐diastolic volume in the control (black) and MR groups (blue). (b1) Decellularized circumferential elastic modulus and (b2) decellularized longitudinal elastic modulus versus end‐diastolic volume in the control (black) and MR groups (blue). Dotted lines represent the best fit line after linear regression analysis. *p*‐values indicate the statistical difference between the slopes of the best fit lines. *p* < 0.05 was considered statistically significant. MR, mitral regurgitation.

**FIGURE 11:**
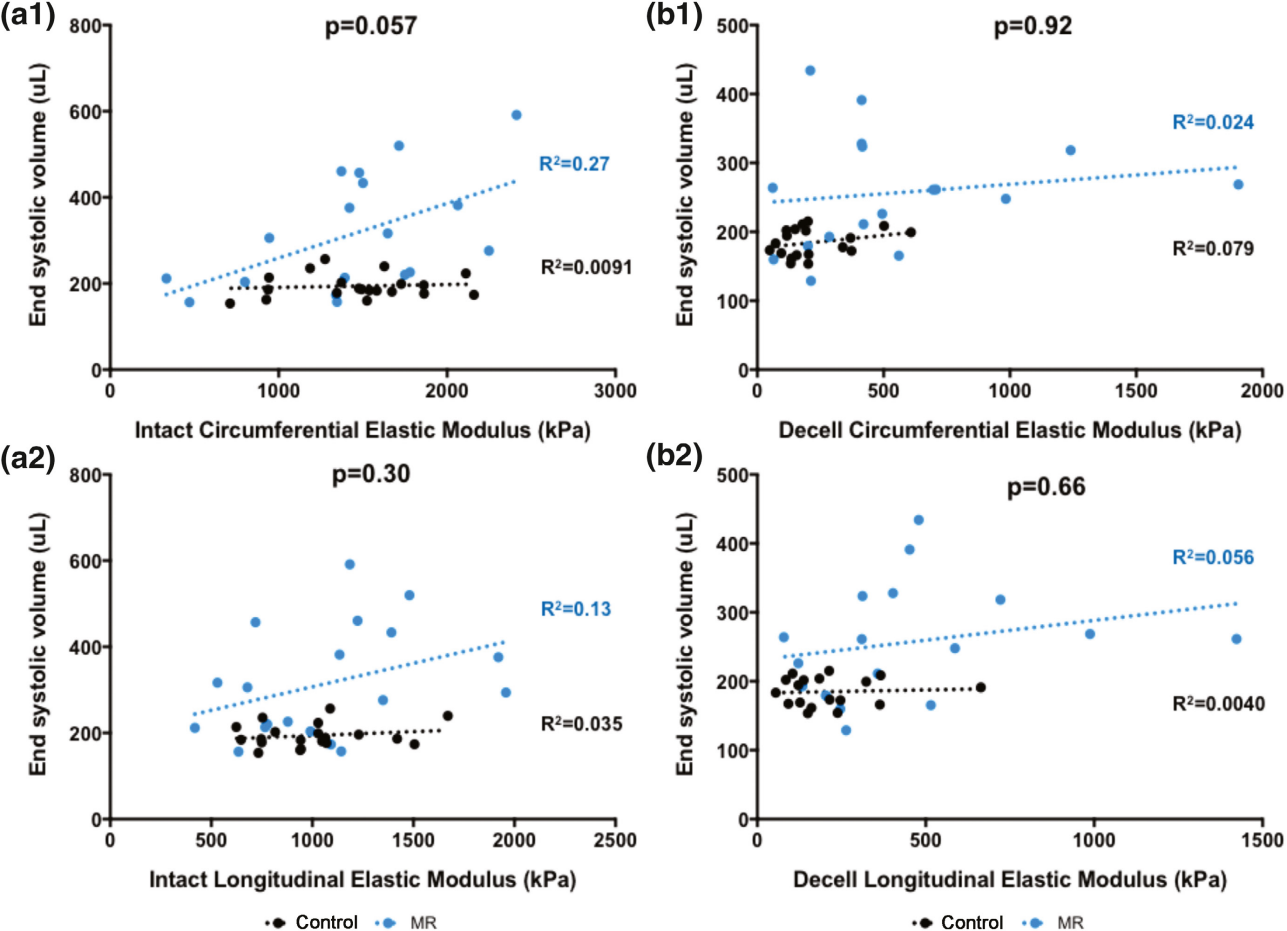
(a1) Intact circumferential elastic modulus and (a2) intact longitudinal elastic modulus versus end‐systolic volume in the control (black) and MR groups (blue). (b1) Decellularized circumferential elastic modulus and (b2) decellularized longitudinal elastic modulus versus end‐diastolic volume in the control (black) and MR groups (blue). Dotted lines represent the best fit line after linear regression analysis. *p*‐values indicate the statistical difference between the slopes of the best fit lines. *p* < 0.05 was considered statistically significant. MR, mitral regurgitation.

The relationship between the elastic moduli of intact and decellularized LV tissues with end‐diastolic volume is shown in Figure 10, and the relationship between the elastic moduli of intact and decellularized LV tissues with end‐systolic volume is shown in Figure 11. For the intact tissues, the circumferential and longitudinal moduli showed a positive linear relationship with end‐diastolic and end‐systolic volumes in the MR group (Figures 10a1–a2 and 11a1–a2). The relationship between the slopes of the circumferential modulus and end‐systolic volume were different in the MR and control groups (*p* = 0.057). For the decellularized LV tissues, the relationship between the moduli and end‐diastolic volume had a slope close to zero in both the control and MR group (Figure 10b1–b2). However, the circumferential and longitudinal moduli showed a positive linear relationship with end‐systolic volume in the MR group with no differences in the slopes between the MR and control groups in the decellularized LV tissues (Figures 10b1–b2 and 11b1–b2).

## 4 DISCUSSION

In this study, we investigated changes to the passive mechanical properties of the left ventricular myocardium in the setting of long‐term MR. Material properties of the intact myocardium and isolated extracellular matrix were quantified. The time‐points 2, 10, 20, and 40 weeks were chosen to reflect the acute and different stages of chronic left ventricular remodeling. The longest time‐point of the study, which is 40 weeks, is equivalent to a duration of twenty human years based on the life‐span of the rat and human (Sengupta, 2013). The age‐ and weight‐matched control animals represented normal myocardium without disease, for comparison.

The intact tissue from control animals exhibited a non‐linear, anisotropic mechanical behavior at every time‐point that was investigated. Decellularizing this tissue and measuring material properties of the isolated matrix exhibited a non‐linear, isotropic response at every time‐point. Thus, in the normal left ventricular myocardium, tissue anisotropy is likely due to the myocyte compartment, and their arrangement within the matrix. In tissues explanted from the MR rats, intact tissue exhibited a non‐linear, anisotropic mechanical behavior as well. In contrast to the control group, the extracellular matrix obtained by decellularization exhibited anisotropy, primarily due to changes in the circumferential direction, with stiffening along this direction. In measuring the changes in the major and minor axis of the LV from ultrasound, we observed that by 40 weeks 30% increase in the major axis and a 20% increase in the minor axis of the LV were observed. The mechanistic basis for such directionality in remodeling is unclear, yet changes in extracellular matrix in the setting of MR are known to occur (Chen et al., 2011; Stewart et al., 2003; Ulasova et al., 2014).

The overall stiffness of the intact ventricular tissue and extracellular matrix did not change with age or weight in the control group. In hearts with MR, gradual stiffening of the myocardium was observed in both circumferential and longitudinal directions. Extracellular matrix material properties also followed a similar trend, with increasing stiffness with time. The ratio of the intact tissue stiffness and the decellularized matrix stiffness was used to investigate the relative contribution of the matrix to overall tissue stiffness. In the hearts with MR, extracellular matrix contributed to only 25% and 33% of the overall tissue stiffness at 2 weeks. By 10 weeks, this relative contribution of the matrix to stiffness increased to 71% and 88% at 10 weeks. When the experimental data were fit to a reduced Fung type strain energy function, the material properties of the decellularized tissue at 20 weeks were significantly higher compared to the control tissue similar to the tangential moduli calculated (Tables S1 and S2). By 20 and 40 weeks, this ratio reduced again to that observed at 2 weeks. We observed that stiffening of the matrix occurs until 10 weeks, and plateaus thereafter. However, a significant rise in the stiffness of the intact tissue occurs along the entire duration, indication potentially a change in the myocyte compartment of the tissue during this entire duration. Recent evidence in different hemodynamic stress states indicates that cardiomyocyte remodeling occurs via cytoskeletal changes which may be driving this stiffening. Within the cardiomyocyte, the passive mechanical properties are governed by titin and the intracellular cytoskeleton (microtubules, intermediate, and actin filaments) (Linke, 2000; Sequeira et al., 2014; Swiatlowska et al., 2020). Alterations to these cytoskeletal proteins in response to stress occurs from detyrosination (Chen et al., 2018), acetylation (Janke & Montagnac, 2017). These phenomenon have been shown to occur in failing cardiomyocytes from chronic VO (Donker et al., 2007). Cytoskeletal breakdown and disorganization of desmin have also been demonstrated in a rodent model (Guichard et al., 2017; Yancey et al., 2015), though the contribution of this feature to overall myocyte stiffness is unclear.

The changes in the passive material properties of the myocardium correlated with a fall in ejection fraction and a rise in end‐systolic volume, in the setting of MR. This observation could have some application in improving current clinical diagnosis of heart failure in MR, using measurement of myocardial stiffness as a modality. Cardiac elastography and wave propagation are two approaches that can be readily implemented clinically. Traditionally, in the setting of VO such as MR, left ventricular dilatation was attributed to increased chamber compliance from extracellular matrix degradation. However, our mechanical testing data does not support this hypothesis, as we do not see increased compliance.

### 4.1 Limitations

As with any experimental study, there are limitations that must be considered when extrapolating this work to the human situation. MR was induced acutely in this model, representing a sub‐set of population only, and not those patients in whom it develops and worsens gradually. Several recent studies have demonstrated genetic mutations that underlie the development of mitral valve prolapse, whose role in the myocardium remains unknown. In this study, we are limited to investigating the effects of VO from MR on normal myocardial tissue, without these valve specific genetic mutations. We use a control group of animals for comparison against the MR experimental groups and not a surgical sham group. From previous experience, we do not see any differences between the geometry or function of the LV of surgical sham rats versus a control group of rats (data not published). However, we do not know whether surgery alone may induce an inflammatory response which would alter the material properties of the LV, especially at the early time‐point of 2 weeks, and thus is a limitation of this study. The decellularized matrix was assumed as a continuum in biaxial studies, whereas in reality, it is a network of fiber bundles, gaps within which can impact the measurement of material properties. Finally, in this study, a simplified exponential function was used to fit the non‐linear stress‐strain data. In future studies, a more complex constitutive model such as the five parameter Gasser‐Ogden‐Holzapel or Humphrey model (Avazmohammadi et al., 2019; Chanda & Callaway, 2018) could be used to improve the biomechanical parameter estimation and characterization of the material response for computational modeling.

## 5 CONCLUSION

In this rat model of MR, we observed that the stiffness of the intact and decellularized left ventricular myocardium increases over time. The relative contribution of the myocyte compartment to the stiffening seems higher than the matrix compartment. Increasing stiffness is also correlated with reduced cardiac function and ventricular dilatation.

## AUTHOR CONTRIBUTIONS

Daniella Corporan and Muralidhar Padala conceived the hypothesis and designed the study. Daniella Corporan. performed the experiments, obtained the data, analyzed the data, and prepared the manuscript. Maher Saadeh, Alessandra Yoldas assisted in experiments and data collection. Jahnavi Mudigonda and Brooks Alexander Lane provided support in data analysis, data modeling and parameter estimation and in preparing some parts of the manuscript. Muralidhar Padala obtained the resources to conduct this work, oversaw the study implementation, prepared and revised the manuscript in collaboration with Daniella Corporan, and approved the final version of this work.

## Supporting information



Supplementary Material
