# Supplementary material for: Passive mechanical properties of the left ventricular myocardium and extracellular matrix in hearts with chronic volume overload from mitral regurgitation
Source: Physiol Rep. 2022 Jul 24;10(14):e15305. doi: 10.14814/phy2.15305 (PMC9309441; doi:10.14814/phy2.15305)
Supplement: Supplementary file 1 — Supplementary Material [file PHY2-10-e15305-s001.docx]

**Supplement**

**Methods:**

The biaxial dataset was analyzed using a reduced Fung type strain energy function, assuming the tissue is incompressible, homogenous, hyperelastic, and neglecting the shear components of the strain tensor. The experimental data was fit to a strain energy function *W* using an exponential Fung model (Sacks M, Biaxial Mechanical Evaluation of Planar Biological Materials, 2000, 61:199-246):

$$W(Q)=\frac{c}{2}\left( e^{Q}-1 \right)\mathrm{EQ}$$

$$Q(E_{ij})= \left( A_{1}E_{11}^{2}+A_{2}E_{22}^{2}+2A_{3}E_{11}E_{22} \right)$$

where *c* and *A_i_* are material constants and *E* is the Green-Lagrange strain. The modeled in-plane 2^nd^ Piola-Kirchoff stress ***S_ij_*** components are derived from the two-dimensional strain energy function *W*:

$\boldsymbol{S}_{\boldsymbol{ij}}=\frac{\partial W}{\partial E_{ij}}$

Individual data sets of 2^nd^ Piola-Kirchoff and Green-Lagrange were fit to the strain energy function by minimizing the objective function:

$e= \sum_{j=1}^{n} \left[ \left( \sigma_{11}^{\mathrm{mod}}-\sigma_{11}^{exp} \right)_{j}^{2}+ \left( \sigma_{22}^{\mathrm{mod}}-\sigma_{22}^{exp} \right)_{j}^{2} \right]$

with fmincon optimizing function in MATLAB, where superscripts *mod* and *exp* refer to model predicted and experimental values of stress with the following constraints:

$$c>0, A_{1}> \left| A_{3} \right|, \mathrm{and} A_{2}> \left| A_{3} \right|$$

**Results:**

None of the parameters in the intact tissue showed any significant differences between the control and MR groups at any time-point. However, in the decellularized myocardium, A2 was significantly higher in the MR group compared to control at the 20wk time-point (p=0.044). This material parameter mathematically is related to the stress-strain relationship in the longitudinal direction. The material parameter, A1, which is mathematically related to the stress-strain in the circumferential was also higher in the MR group compared to sham decellularized myocardium at 20wks, however the values were not statistically significant (p=0.678). These material parameters estimated from fitting the experimental data to this constitutive model reflect the tangential modulus in both the circumferential and longitudinal directions which was calculated from the experimental data and shown in Figure 8.

**Supplement Table 1. Intact myocardial parameter estimation from the simplified Fung type strain energy function.**

|  | c | | | A1 | | | A2 | | | A3 | | |
| --- | --- | --- | --- | --- | --- | --- | --- | --- | --- | --- | --- | --- |
|  | **Control** | **MR** | **p-value** | **Control** | **MR** | **p-value** | **Control** | **MR** | **p-value** | **Control** | **MR** | **p-value** |
| 2wk | 4.44 ± 1.45 | 2.39 ± 1.14 | >0.999 | 42.12 ± 11.55 | 36.13 ± 14.87 | >0.999 | 21.51 ± 10.41 | 20.61 ± 8.66 | >0.999 | 4.31 ± 1.66 | 3.81 ± 1.39 | >0.999 |
| 10wk | 1.55 ± 0.38 | 4.52 ± 4.72 | >0.999 | 61.45 ± 17.92 | 45.17 ± 28.25 | >0.999 | 31.64 ± 10.84 | 18.63 ± 9.58 | 0.539 | 6.31 ± 1.88 | 4.00 ± 2.13 | >0.999 |
| 20wk | 2.97 ± 2.48 | 4.71 ± 3.31 | >0.999 | 75.07 ± 19.94 | 73.34 ± 34.16 | >0.999 | 27.42 ± 12.25 | 27.78 ± 10.31 | >0.999 | 6.07 ± 2.04 | 6.02 ± 2.368 | >0.999 |
| 40wk | 4.44 ± 1.45 | 3.03 ± 1.60 | >0.999 | 42.12 ± 11.55 | 48.77 ± 15.50 | >0.999 | 21.51 ± 10.41 | 21.13 ± 6.72 | >0.999 | 4.31 ± 1.66 | 4.33 ± 1.06 | >0.999 |

**Supplement Table 2. Decellularized myocardial parameter estimation from the simplified Fung type strain energy function.**

|  | c | | | A1 | | | A2 | | | A3 | | |
| --- | --- | --- | --- | --- | --- | --- | --- | --- | --- | --- | --- | --- |
|  | **Control** | **MR** | **p-value** | **Control** | **MR** | **p-value** | **Control** | **MR** | **p-value** | **Control** | **MR** | **p-value** |
| 2wk | 2.94 ± 2.04 | 2.42 ± 3.90 | >0.999 | 11.32 ± 10.24 | 21.67 ± 12.40 | >0.999 | 13.47 ± 13.59 | 18.85 ± 16.05 | >0.999 | 1.79 ± 1.75 | 2.82 ± 1.80 | >0.999 |
| 10wk | 1.36 ± 0.65 | 0.50 ± 0.43 | >0.999 | 13.27 ± 9.22 | 50.01 ± 19.92 | >0.999 | 10.38 ± 3.91 | 30.67 ± 18.01 | 0.807 | 1.63 ± 0.65 | 5.18 ± 0.95 | >0.999 |
| 20wk | 12.77 ± 11.90 | 15.34 ± 26.23 | >0.999 | 6.01 ± 3.42 | 45.33 ± 50.79 | 0.678 | 3.73 ± 2.31 | 50.82 ± 58.98 | 0.044 | 0.61 ± 0.36 | 6.57 ± 7.22 | 0.344 |
| 40wk | 16.93 ± 29.18 | 2.67 ± 0.74 | >0.999 | 10.51 ± 13.48 | 19.98 ± 16.82 | >0.999 | 7.30 ± 5.43 | 12.71 ± 11.08 | 0.999 | 1.22 ± 1.20 | 1.93 ± 1.16 | >0.999 |
